# Pharyngeal and Nasal Surgery by the Galvano-Cautery

**Published:** 1886-10

**Authors:** W. E. Casselberry

**Affiliations:** Professor of Materia Medica and Therapeutics and of Laryngology and Rhinology, Chicago Medical College; 70 Monroe Street


THE CHICAGO

### MEDICAL JOURNAL AND EXAMINER.

VOL. LIII. OCTOBER, 1886. No. 4.

### ORIGINAL COMMUNICATIONS.

PHARYNGEAL AND NASAL SURGERY BY THE GALVANO-CAUTERY, WITH A REPORT OF CASES. *By* W. E. CASSELBERRY, M.D., *Professor of Materia Medica and Therapeutics and of Laryngology and Rhinology, Chicago Medical College.*

[Read before the Chicago Medical Society, September 20.]

New methods of utilizing the galvano-cautery are constantly being devised. Both the battery and electrodes have recently been so improved as to materially extend its field of usefulness, and the discovery of the local anæsthetic, hydrochlorate of cocaine, has been of inestimable benefit in the same direction. Its applications, if properly managed, are attended with little or no pain, it is rapid in action and satisfactorily thorough, oftentimes brilliant in its results. In nasal, pharyngeal and even in laryngeal surgery its utility is particularly manifest. No other means has so augmented the remedial powers of the rhinologist. Pathological conditions of the upper respiratory passages which have long baffled the most skillful therapeutists are now effectually remedied through the medium of this agent.

The few cases herewith reported are grouped together only because of the similarity in the means of treatment, although each case is of interest from a pathological as well as from a therapeutic point of view.

CASE I.—*Folliculous Pharyngitis with Hypertrophy in the Angles of the Pharynx.*

Doctor —— has suffered for an indefinite period from fullness, soreness and pain in the pharyngeal region. He has frequent attacks of acute pharyngitis, and at such periods of exacerbation, his sense of hearing is affected, evidently by extension of the inflammatory process through the Eustachian orifices, a common sequence, and one of especial moment in his case.

*Status Præsens.*—By reflected light the oro-pharynx is seen to be intensely congested, and three or four isolated follicular enlargements appear on the posterior pharyngeal wall ; but most conspicuously prominent are two enormous hypertrophied follicular masses which occupy the latero-posterior angles of the pharynx. These masses, one on each side, jutted inward and forward from behind the posterior pillar, and were of the thickness of a lead-pencil and about three-fourths of an inch in length, each with the adjoining tonsil presented the appearance of a double tonsil with the parts separated by the posterior palatine fold.

In the normal state, isolated follicular glands, called muco-lymphoid follicles, are imbedded in the pharyngeal wall. Each in its simplest form consists of a depression of the mucous membrane lined with its epithelium, and enveloped in a stratum of reticular connective-tissue, entangled in which are innumerable lymphoid cells, lymphoid bodies (closed follicles) and lymphatic and other vessels. Morphologically, they are of the same structure as the tonsils, the latter representing large aggregations of such simpler glands. Hypertrophy of these muco-lymphoid follicles is the chief pathological condition characterizing folliculous pharyngitis. The same changes take place, which occur in the tonsil when it is in a state of chronic hypertrophy. The inflammatory process leading to cell-proliferation involves every structural element of the gland; its secretion is retained, accumulates and undergoes degeneration, and the follicle thus metamorphosed becomes a center of irritation into which course numerous enlarged blood-vessels, which serve to maintain continued congestion of the parts. Now a whole chain of these muco-lymphoid follicles ranges along in each latero-posterior angle of the pharynx, behind the posterior pillar, and it is the hypertrophy of this entire row of glands on each side which is the chief feature in the case now under consideration. Although rarely as enormous as in the present instance, hypertrophy in this particular locality is not uncommon, but it is usually overlooked or regarded as of too little consequence to necessitate treatment of an active nature.

*Treatment.*—Destruction of the hypertrophied follicular masses was evidently the only effective remedy, and the galvano-cautery suggested itself as the means best adapted to the end. I extemporized an electrode for the purpose by uncoiling the wire end of a Flemming moxa-electrode, thus converting the platinum coil into a loop three-fourths of an inch long, with two parallel side wires one-eighth of an inch apart. This was so curved as to be capable of accurately approximating the mass.

It was applied first cold, pressed firmly against the part and the battery then connected. Instantly the loop became white hot, and produced its slough. By breaking the current, it at once became cool and was ready for withdrawal. It caused very little pain, no hæmorrhage, and but slight subsequent irritation. Several operations were necessary to entirely destroy the growths. They were performed alternately on the two sides, always allowing sufficient interval between operations on the same side for separation of the slough and cicatrization.

The few isolated follicular enlargements on the posterior wall of the pharnyx were destroyed in like manner by the Flemming point-electrode.

The result is highly satisfactory. Nearly a year has elapsed since the operations, and the pharynx remains free from hypertrophies.

In this connection I would note that topical applications, in cases of chronic folliculous pharyngitis, of medicaments other than those which affect destruction of the individual growths, are futile and oftentimes worse than useless.

CASE II.—*Folliculous Naso-Pharyngitis*, *or Adenoid Vegetation* at the vault of the pharynx. Operations by the Galvano-Cautery Ecraseur.

Miss —— has complained of catarrhal disease of the upper respiratory passages involving the nose, naso-pharynx and larynx. Purulent nasal and pharyngeal discharges, hawking screatus, cough, hoarseness and a nasal intonation are the chief symptoms. She takes cold easily and frequently, and at such times suffers exacerbation of all symptoms. Examination, in addition to morbid conditions of other parts, revealed numerous hypertrophied follicular masses—so called adenoid vegetations—covering and filling up the vault of the pharynx. Some of these appeared in the rhinoscopic mirror as pendent pear-shaped bodies; others were sessile, and still others had the appearance of several cockscombs crowded together. There is nothing unusual thus far about the case. Its pathology has been many times described, and I will review it but briefly, as it is to the method of operating that I desire especially to invite attention.

Normally, muco-lymphoid follicles, of the same adenoid structure exactly as those in the pharynx, are imbedded in the mucous and sub-mucous tissues of the naso-pharynx. A number of these are grouped together, forming a compound gland, analogous to the tonsils and known as the pharyngeal tonsil, or the tonsil of Luschka. In the normal state this is scarcely of sufficient size to deserve such appellation, but when hypertrophied, as it frequently is, it bears a striking resemblance to the true tonsil. The tonsil of Luschka and the surrounding isolated muco-lymphoid follicles, like the analogous structures in the oro-pharynx, are exceedingly prone to undergo the hypertrophic process, attaining at times an enormous development. This constitutes one form of post-nasal catarrh, the general symptoms of which are so familiar as to render it unnecessary to enumerate them.

*Treatment.*—Removal of the hypertrophied masses is the only satisfactory means of relief.

This I first sought to accomplish in the present case with a Loewenberg cutting forceps, an instrument especially designed for the purpose, but the effort resulted unsatisfactorily. Several other methods were tried and abandoned, until finally I found that the object could be attained most easily, and with the least pain and inconvenience to the patient, by means of a galvano-cautery ecraseur.

It was necessary to construct an ecraseur for the case, which I did by first bending a probe to exactly the proper curve to pass behind the *velum palati* and touch the hypertrophied masses, and then making a pair of Flemming's parallel tubes to conform to the same curve, and threading them with platinum wire to form the loop. Withdrawing the soft palate by a Voltolini palate-hook, the naso-pharynx could be thoroughly sprayed with cocaine solution, which not only rendered the operation painless, but entirely prevented the retraction of the velum consequent upon a disposition to gag, so that a perfect rhinoscopic image of the parts could at all times be obtained; that is, I could see exactly what I was doing, even during the snaring.

The patient controlled her own tongue by means of Türck's tongue-depressor. Guided by the rhinoscopic image I introduced the ecraseur and caused it to encircle one of the growths, and at the same time, by tightening the wire somewhat, to grasp it firmly. Then, by connecting the battery, the loop was heated instantly to redness. It was rapidly wound in on the windlass and the vegetation thus severed at its base. There was no pain, no hæmorrhage, and but little subsequent irritation. The operations were repeated weekly for a number of weeks, until the growths were entirely removed.

CASE III.—*Malformation of the Anterior Pillars of the Fauces.*

Mr. —— has complained for some years of a sense of constriction in the pharynx, as if his throat were too small, and also of the habit of snoring. The latter is so loud and so frequent as to be a nuisance to his wife and family, and a disturbance to himself, inasmuch as he is often awakened at night by his own noise.

On inspecting the parts I was first impressed by the very small size of his throat, in comparison with the rather broad dimensions of the individual, including his neck and head.

I have often noted the real narrowness of the pharynx in fleshy persons, and I attribute it to a deposit of adipose material in and between the pharyngeal muscles to such an extent as to encroach on the free space within. This would explain the increase in the severity of Mr. ——'s symptoms, as he has gradually grown stouter. The tonsils were somewhat hypertrophied, but what was of still more importance there was found to exist a malformation of the anterior pillars of the fauces, *i. e.*, of the palato-glossal folds. These pillars are formed by the palato-glossal muscles, inclosed by connective-tissue and mucous membrane. Normally the muscle proceeds each from the anterior and lateral portion of the *velum palati*, sweeps over the anterior edge of the tonsil and, extending forward, is attached together with the stylo-glossus muscle to the side of the tongue near its root. In this case the folds originated properly, but increasing in breadth they covered not only the anterior edge of each tonsil, but also a considerable portion of its inner surface, and then, a part only of the fold continuing in the normal pathway forward to the side of the tongue, the larger portion swept *backward* and incorporated itself with the posterior pillar, forming a thick band which continued downward in the latero-posterior angle of the most inferior portion of the pharynx.

In their normal relations the palato-glossal folds assist in drawing the velum forward, and so aid in maintaining sufficient space between it and the post-pharyngeal wall for free and noiseless respiration through the nose.

Their abnormal attachment and development tended to destroy this balance of muscular power, and, as a result, the post-palatine space was so contracted that the air-current passing to and fro, especially during sleep and in the recumbent posture, caused forcible vibration of the soft palate, or snoring.

*Treatment.*—I determined to excise the overgrowth of each tonsil, together with the portion of the anterior pillar which covered it, by means of the galvano-cautery ecraseur, and at the same time to sever, as low down as possible, the attachments of the anterior pillars to the pharyngeal wall.

Piano wire No. 5, a steel or tempered iron wire, replaced the usual platinum coil in the ecraseur, because its resiliency, a property totally wanting in platinum, permitted a better adaptation of the loop to the mass. Having sprayed the parts thoroughly with a four per centum cocaine-solution, I first nicked with scissors the muscular band at its attachment to the posterior wall, in order that the wire could catch hold at that point. Then encircling the part by the loop of the ecraseur and connecting the battery, the wire at once was heated to a cherry-red. It was rapidly wound in and the tissue was thus removed.

After cicatrization had occurred a similar operation was performed on the other side.

The result is very satisfactory. The configuration of his pharynx approximates the normal; the sense of constriction has disappeared, and the habit of snoring, although not entirely cured, is much relieved.

CASE IV.—*Membranous Occlusion of the Posterior Nares.*

This case I reported at length in a paper read before the Chicago Medical Society a year ago. I briefly review it, now, because of its special interest in this connection, and to report permanence of cure, and progress in the almost complete alleviation of all catarrhal symptoms which was unhoped for at that time.

Mr. E., age about 40, has suffered during the past thirteen years from a complete obstruction of the left nasal chamber. Most violent paroxysmal headaches, constant soreness on top of the head, vertigo, especially on stooping, and various indescribable cephalic sensations of a most distressing character, have served to render his life miserable.

Thick, viscid and foul muco-purulent crusts accumulated in the left naris and in the naso-pharyngeal space, which he could neither expectorate nor evacuate by "blowing the nose," on account of the impenetrability of the nostril to currents of air. Partial occlusion, also, of the right nasal chamber necessitated frequent and prolonged resource to mouth-breathing, and he has suffered in consequence from atrophic pharyngitis and laryngitis, with painful deglutition, cough, severe asthmatic paroxysms, and the like. The expired air had imparted to it a disgustingly fetid odor. During all these years he has been treated for consumption, bronchitis, catarrh, neuralgia, rheumatism, and other complaints,—a rhinoscopic examination never having been made. He was referred to me by my friend Dr. R. H. Babcock, of this city.

It was only after some days' cleansing of the parts and training of the patient that I could get a perfect rhinoscopic image. It was found that a tense membrane covered the left choana almost completely. Its free edge was thin and sharp and approached so near to the septum that, as seen in the drawing, only a very small chink remained between it and the *septum narium.* The left *ostium tubæ* could not be seen, the membrane evidently lying behind the Eustachian orifice, and so intercepting its image. The choana upon the right, also, was partially covered by a membrane which extended about half way across the aperture and which was much thicker and less tense than that upon the left side.

*Treatment.*—The galvano-cautery knife-electrode at once occurred to me as the most effective means of removing these membranous obstructions.

The patient was practiced daily for one week in the use of the rhinoscope and in the introduction of instruments. A flexible silver probe was bent by numerous trials to exactly the proper curve to pass readily, through the mouth and naso-pharynx, into the chink on the left side and to pick up the edge of the membrane, and then a straight knife-electrode was made to conform to the same curve.

*First Operation.*—The patient held his own tongue by means of Türck's tongue-depressor. I introduced the knife-end of the electrode, one-half inch long, through the chink and pressed its edge against the membrane, backward. The current was made at the battery, the knife end became in an instant red hot and the membrane was incised without bleeding and without sufficient pain to cause the patient to wince. In another instant the current was broken and the electrode cooled in a few seconds and was withdrawn.

After incision the membrane, which had been very tense at its edge, retracted from each side, leaving a surprisingly large opening.

The delight of the patient, when he discovered that he could again breathe through that nostril, was a pleasure to behold, and from that moment he has been completely relieved of all cephalic symptoms, including the left sided deafness.

I found the floor of the nose, the septum and the turbinated bodies thickly incrusted with horribly fetid cheesy masses, the accumulated, decomposed and desiccated secretions of years.

These were so firmly adherent that they could be detached only by means of a probe during a period extending over several days.

Their presence, for so many years, had served to excite a bad naso-pharyngeal catarrh of an atrophic type, which at that time I had little hope of benefiting, but as an illustration of the recuperative powers of nature, even this complication, in the absence of the original cause and under favorable treatment extending over several months, has so far improved as to occasion, practically, no annoyance.

Additional operations, to the number of seven or eight, were performed in exactly the same manner, now on one side and then on the other, until both posterior nares were widely patent and in an approximately natural condition.

These are but a few of many cases wherein I have found the galvano-cautery of the utmost utility. Doubtless the several operations could have been performed by other means, but not without procedures so much more formidable that both the surgeon and patient would have preferred to forego operation.

Certainly this agent can be abused, and a word of caution is not unnecessary in this respect. There is reason to believe that injurious effects have already been produced by it, especially in the nasal regions. Morell Mackenzie warns some of his younger *confrères*, "that it is unnecessary, where no inconvenience is felt, to restore geometrical symmetry to the turbinated bodies or to invest the lining membrane of the nose with artistic merit," and he fears "that the nasal passages have occasionally been 'cleared' with a zeal and energy worthy of the industrious backwoodsman." But while deprecating unnecessary aggression in these regions, he at the same time asserts that there are many cases that can only be cured by active treatment. Accurate diagnosis, precise indications for its use, and a certain amount of skill on the part of the operator, are essentials to the judicious employment of the galvano-cautery.

The battery and electrodes which I employ, and which I take pleasure in exhibiting to the Society, are manufactured by Otto Flemming, of Philadelphia, from suggestions furnished by Carl Seiler. The battery has advantages over any other that I have seen. Not the least of these is the arrangement by which the plates may be immersed and withdrawn from the fluid by means of a pedal easily manipulated by one foot. Electrodes can be devised and altered from the standard ones to suit individual cases.

70 Monroe Street.

